# Privacy-Preserving Detection of Tampered Radio-Frequency Transmissions Utilizing Federated Learning in LoRa Networks

**DOI:** 10.3390/s24227336

**Published:** 2024-11-17

**Authors:** Nurettin Selcuk Senol, Mohamed Baza, Amar Rasheed, Maazen Alsabaan

**Affiliations:** 1 Department of Computer Science, Sam Houston State University, Huntsville, TX 77340, USA; nss016@shsu.edu (N.S.S.); axr249@shsu.edu (A.R.); 2 Department of Computer Science, College of Charleston, Charleston, SC 29424, USA; 3 Department of Computer Engineering, College of Computer and Information Sciences, King Saud University, P.O. Box 51178, Riyadh 11543, Saudi Arabia; malsabaan@ksu.edu.sa

**Keywords:** LoRa networks, privacy, security, IoT, federated learning

## Abstract

LoRa networks, widely adopted for low-power, long-range communication in IoT applications, face critical security concerns as radio-frequency transmissions are increasingly vulnerable to tampering. This paper addresses the dual challenges of privacy-preserving detection of tampered transmissions and the identification of unknown attacks in LoRa-based IoT networks. Leveraging Federated Learning (FL), our approach enables the detection of tampered RF transmissions while safeguarding sensitive IoT data, as FL allows model training on distributed devices without sharing raw data. We evaluated the performance of multiple FL-enabled anomaly-detection algorithms, including Convolutional Autoencoder Federated Learning (CAE-FL), Isolation Forest Federated Learning (IF-FL), One-Class Support Vector Machine Federated Learning (OCSVM-FL), Local Outlier Factor Federated Learning (LOF-FL), and K-Means Federated Learning (K-Means-FL). Using metrics such as accuracy, precision, recall, and F1-score, CAE-FL emerged as the top performer, achieving 97.27% accuracy and a balanced precision, recall, and F1-score of 0.97, with IF-FL close behind at 96.84% accuracy. Competitive performance from OCSVM-FL and LOF-FL, along with the comparable results of K-Means-FL, highlighted the robustness of clustering-based detection methods in this context. Visual analyses using confusion matrices and ROC curves provided further insights into each model’s effectiveness in detecting tampered signals. This research underscores the capability of federated learning to enhance privacy and security in anomaly detection for LoRa networks, even against unknown attacks, marking a significant advancement in securing IoT communications in sensitive applications.

## 1. Introduction

The rapid growth of the Internet of Things has spurred widespread adoption of Long Range (LoRa) networks, a wireless communication technology that supports low-power, long-range connectivity, making it highly suitable for IoT devices. LoRa’s capability to transmit data across extended distances while conserving energy is precious in industrial automation, smart cities, and agriculture. However, as LoRa networks in IoT use grow, these systems become more susceptible to security threats, including manipulating RF communications. Adversaries can exploit LoRa networks through jamming, eavesdropping, and man-in-the-middle (MitM) attacks, which can significantly compromise critical applications [1].

### 1.1. Challenges

Securing LoRa networks introduces unique challenges due to the limited computational resources of these low-power devices, which often restricts the feasibility of traditional, resource-intensive security mechanisms. Anomaly detection has emerged as a promising approach to identifying malicious RF signals by recognizing deviations in transmission patterns that suggest tampering. One-Class classifiers, such as One-Class Support Vector Machines (OCSVMs), are especially suited for this task as they require only normal (untampered) data for training, making them ideal for identifying unknown or previously unseen attacks. These models learn the patterns of normal transmissions, enabling the detection of anomalies—an approach that is particularly advantageous for LoRa networks where data storage and processing capacity are constrained.

While effective, many existing anomaly-detection systems rely on centralized data aggregation, where raw data are transmitted to a central server for analysis. This centralized approach can compromise data privacy, especially when IoT devices contain sensitive information.

Federated learning (FL) solves these privacy challenges by allowing IoT devices to collaboratively train models without sharing raw data. In FL, each device trains a model locally on its data and only shares model updates rather than the raw data itself, with a central server that aggregates these updates into a global model. This decentralized approach reduces data transmission and minimizes privacy risks, making it particularly compatible with resource-constrained LoRa networks. Through federated learning, LoRa networks can detect tampered transmissions while preserving the privacy of sensitive device data, addressing both security and privacy concerns [2].

For instance, in industrial IoT applications, frequency data can expose operational schedules or machinery patterns, while in smart cities, it could reveal user-specific routines like commuting or energy usage. By leveraging FL, these private details remain locally protected, as the central model only aggregates patterns across devices without collecting individual device data. This decentralized approach significantly reduces the risk of privacy leakage, ensuring that frequency tampering or anomalies can be detected without compromising user confidentiality.

### 1.2. Contributions

This paper introduces a privacy-preserving method for detecting tampered RF emissions in LoRa networks by combining federated learning with one-class classifiers and other advanced anomaly-detection techniques. Our approach leverages FL’s privacy benefits, allowing LoRa devices to identify anomalies in RF transmissions, such as frequency shifts, packet tampering, and signal manipulation, without exposing sensitive data. The key contributions of this work include:

1. The use of federated learning to preserve privacy in IoT networks, employing One-Class Classifiers to detect tampered RF frequencies and validating these methods through experiments. Using FL ensures that sensitive data remain on local devices, with only model updates shared.
2. Employing One-Class Classifiers to detect tampered RF frequencies and validating these methods through experiments. By using FL, we ensure that sensitive data remain on local devices, with only model updates shared through the use of federated learning to preserve privacy in IoT networks.
3. Using One-Class Classifiers like OCSVM allows for efficient detection of tampered signals, while the FL approach optimizes accuracy across multiple devices. The experimental results demonstrate that the proposed FL-based method enhances privacy protection and achieves high accuracy in detecting tampered RF transmissions, outperforming traditional centralized machine-learning approaches.

Through rigorous testing, we assessed various anomaly-detection models within the federated learning framework, including OCSVM, Convolutional Autoencoder (CAE), Isolation Forest (IF), Local Outlier Factor (LOF), and K-Means. Our results demonstrate that federated one-class classifiers can achieve high accuracy, precision, recall, and F1-scores, with the top-performing models surpassing 97% accuracy. These findings highlight the efficacy of a federated learning-based approach in securing IoT networks, particularly in scenarios where data privacy is critical.

### 1.3. Paper Organization

The remainder of this article is organized as follows: Section 2 reviews prior research on anomaly detection in LoRa networks. Section 3 outlines the system and threat models that guide this paper. Section 4 details the proposed framework, followed by Section 5, which discusses the evaluation metrics. Section 6 presents and analyzes the results. Finally, Section 7 summarizes key findings, addresses limitations, and suggests directions for future research.

## 2. Literature Review

With a background in identifying tampered radio-frequency communication, this section of our paper examines the most recent security research in the IoT, LoRa technology, and FL. A critical discussion of earlier research on this subject will be conducted to determine the advancements gained and the knowledge gaps. By examining several academic publications, technical reports, and case studies, this review provides a tangible background knowledge of the context and importance of our investigation. Critical issues and emerging developments in the field include secure communication in LoRa-based IoT networks and privacy-preserving anomaly detection. The review has discussed various research issues while pointing at federated learning as an enabler of decentralized, secure detection with data privacy preservation and protection against RF tampering of IoT networks in a more connected world.

The work in [3] proposes a system for federated learning on microcontroller-based IoT devices using a LoRa mesh network. A dual-board setup was implemented, where the Arduino Portenta H7 manages a neural network for keyword spotting, while the TTGO LORA32 board operates as a network router. By integrating machine learning and LoRa communication, the system demonstrated that federated learning can be feasible even on resource-constrained devices. Performance analysis covered model accuracy, bandwidth, and energy consumption, revealing that while LoRa-enabled federated learning supports on-device training and distributed updates, it comes with longer training times due to LoRa’s limited data rate. Potential for bandwidth efficiency was explored through optimized neural network weight representation, showing bandwidth savings but highlighting a need for further improvements. This work underscores LoRa’s viability in federated learning but suggests that adaptive updates and energy-efficient optimizations will be essential for future implementations. Future research should evaluate other machine learning applications to refine system design and address the balance between model accuracy gains and resource costs in real-world deployments.

The paper [4] presents FedLoRa, an optimization framework designed to enhance federated machine learning (FML) in LoRa-based IoT networks. The study demonstrates that FML can still converge even with communication losses up to a certain threshold, a critical finding for resource-limited IoT environments. FedLoRa optimizes network load by balancing the spreading factors and utilizing sequential polling of nodes to maximize non-interfering transmissions, reducing FML round times without increasing energy consumption. The problem is formalized as the LoRa Resource Allocation Problem (LoRa-RAP), and an approximation algorithm is provided to solve it efficiently. Experimental results, including tests on the **Colosseum channel emulator and real-world measurements in Portland, Maine, show that FedLoRa can reduce FML round times by up to 35% compared to baseline methods. The framework also leverages the insight that learning errors can be minimized by adjusting the learning rate across rounds. The authors pledge to share their code, ensuring the reproducibility of the results, and positioning FedLoRa as a significant advancement in optimizing FML for IoT networks.

This work [5] addresses key challenges in applying machine learning (ML) to Internet of Things (IoT) applications, focusing on privacy preservation and minimizing communication costs. The study presents a novel framework for anomaly detection of ECG signals from low-cost wearable sensors, transmitted via LoRaWAN, a low-power wide-area network (LPWAN) technology. The framework utilizes federated learning (FL) to maintain user data privacy while optimizing communication between end devices and the gateway. The proposed system significantly reduces data transmission requirements, achieving a 98% reduction in data volume compared to traditional centralized ML methods, without compromising performance. The framework’s effectiveness is demonstrated through computer simulations, highlighting its potential for efficient, privacy-preserving IoT applications.

Federated learning with privacy protection has received much attention lately [6,7,8,9]. A privacy-enhanced FL framework was created by Hao et al. [10] to encourage efficiency and privacy in FL operations. FL was used by Dolui et al. [11] to ensure the privacy and functionality of matrix factorization and recommender systems. White-box inference attacks were the topic of a thorough privacy analysis by Nasr et al. [12]. A system that combines generative adversarial networks and a multitask discriminator was presented by Wang et al. [13] to reduce user-level privacy leaks in FL and defend against malicious server assaults.

Several federated learning (FL) frameworks have been discussed in the past works to address data confidentiality issues [14,15,16,17,18,19,20]. These frameworks share trained models with participants rather than raw datasets, guaranteeing data confidentiality and utility while preserving privacy. To protect against poisoning attacks, Qu et al. [21] created a completely decentralized FL framework that uses blockchain technology and the Proof-of-Work (PoW) consensus mechanism. Lu et al. [22] presented an architecture that combines a permissioned blockchain system with deep learning (DL) to solve data-sharing issues. By using encrypted logs kept up to date by central IoT nodes, this architecture creates safe connections between Internet of Things (IoT) devices at the network edge, guaranteeing data availability and confidentiality. Instead of storing raw data, the framework uses a permissioned blockchain to retrieve relevant data and control access, effectively solving storage and privacy issues.

Briggs et al. studied privacy-preserving techniques for FL in the context of the Internet of Things, highlighting the challenges that arise in resource-constrained environments. Hu et al. focused on using personalized models on each device to implement differential privacy (DP) in FL by leveraging smartphone activity recognition data. Lu et al. also used blockchain for the consensus mechanism, where computational resources are also used for federation training. However, they did not provide evaluation results specific to the DP technique. Zhao et al. studied the effects of Gaussian noise as a DP approach in FL; however, their evaluation was limited to the MNIST dataset and did not compare alternative DP strategies. Additionally, Hu et al. [23] investigated a resource-constrained IoT scenario and implemented and evaluated a more relaxed version of DP across different datasets.

One of the critical developments in federated learning is the development of Privacy-Preserving Federated Learning frameworks, which provide efficient model training while safeguarding user privacy. The privacy-focused PPFL architecture, which first presented [24], uses TEEs to perform safe aggregations of model updates and local training. Therefore, the proposed approach will prevent adversaries from knowing sensitive information from tampered transmission, making the LoRa network particularly suitable in data integrity applications. They also proposed the xMK-CKKS scheme, wherein model updates are encrypted before sharing with a central server by [25]. This scheme enhances the security of collaborative learning processes by addressing privacy leakage risks associated with sensitive radio-frequency data. In addition, the system’s privacy is improved because, for decryption, participating devices have to collaborate so that even when multiple devices are compromised, the security of tampered transmission detection remains intact. An asynchronous FL framework designed explicitly for multi-UAV-enabled networks was developed by [26], enabling local model training without transferring raw data. For multi-UAV-enabled networks, Ref.  [26] created an asynchronous FL framework that allows local model training without sending raw data. This is pertinent given that devices on LoRa networks are susceptible to manipulation. What makes it unique is that it uses a device selection approach that eliminates subpar devices, guaranteeing that only high-quality data reliably contributes to the model during the learning process.

Generally speaking, communication in federated learning should be efficient; this requirement amplifies when working over resource-constrained environments, such as LoRa networks. Ref. [27] proposes the FTTQ algorithm, which significantly reduces the communication cost by reducing the amount of data sent to the server without causing any degradation in model performance. This is an essential optimization for the timely detection of tampered transmission to improve radio-frequency signal monitoring further.

The work in [28] proposed a distillation-based federated learning approach that resolves some of the challenges in communications and puts a simultaneous focus on protecting privacy. In this technique, the robustness of the detection mechanisms is enhanced because only sensitive radio-frequency information needs to remain private. At the same time, knowledge distillation among the local models takes place effectively. As such, it becomes pretty suitable for LoRa networks while handling operational environments of diverse nature.

In detecting manipulated transmissions, federated learning and blockchain technology offer a new method for secure model upgrades. Awan et al. [29] developed a blockchain-based privacy-preserving federated learning (BC-based PPFL) system using the decentralized trust features of blockchain technology. By enhancing the security of shared model updates through federated learning, this framework preserves the integrity of LoRa network identification techniques.

The heterogeneous nature of data in the IoT contexts complicates the federated learning process and often leads to Identically Distributed (non-IID) and non-independent problems. An AIoT plan with federated learning capabilities that prioritizes data exchange while retaining privacy was developed by Su et al. [30]. In order to accurately identify tampered signals in LoRa networks and enhance the learning process’s reliability, their methodology considers non-IID data distributions.

Furthermore, Li et al. [31] presented the chain-PPFL framework, which uses a single-masking and chained-communication approach to secure sensitive data during federated learning. This feature permits safe model update sharing without jeopardizing underlying data privacy and is crucial for creating reliable detection techniques for manipulated transmissions.

Senol et al. [32] address the significant challenge of detecting tampered radio-frequency signals in LoRa networks, widely used in IoT applications for their long-range, low-power capabilities. Despite LoRa’s advantages, these networks are highly susceptible to interference and signal manipulation, compromising data integrity and security. Senol et al.’s innovative solution uses five anomaly-detection algorithms—Principal Component Analysis, Variational Autoencoder, Isolation Forest, Local Outlier Factor, and a traditional Autoencoder—that apply image-based techniques to identify frequency alterations in LoRa-based IoT systems. Their approach creates a dataset of over 26,000 images by decomposing both normal and tampered LoRa signal transmissions into individual frames, allowing a comprehensive evaluation of each algorithm. Their findings show Local Outlier Factor achieved the highest detection accuracy at 97.78%, followed by Variational and traditional Autoencoders, Principal Component Analysis, and Isolation Forest with 97.27% and 84.49% accuracy, respectively.

However, this approach presents two primary challenges: privacy and the detection of zero-day attacks. The reliance on centralized image-based anomaly-detection methods means sensitive data are processed in a shared or central location, raising privacy concerns. For instance, frequency patterns can reveal details about device usage, operational schedules, or user behavior, which could inadvertently expose confidential information. Moreover, detecting zero-day attacks—previously unseen or unknown signal tampering—remains challenging. Anomaly detection models trained on known tampering patterns may struggle to identify these new, subtle forms of interference or manipulation. Addressing these issues would require privacy-preserving techniques, such as federated learning and adaptive, robust anomaly-detection algorithms capable of generalizing to unseen attack types, enhancing LoRa network security while protecting user privacy.

Ref. [33] addresses the challenge of secure authentication in LoRa networks, especially in IoT and intelligent vehicular systems, where traditional methods struggle with network heterogeneity and chirp signal variability. The authors propose a Deep Federated Learning Network (DFLNet) to leverage radio frequency fingerprinting (RFF) for decentralized, privacy-preserving authentication. Using mobile edge computing (MEC) servers and an adaptive optimizer, DFLNet ensures efficient learning across diverse networks. Empirical results show DFLNet achieves 96.7% accuracy with just 5000 samples per client, offering a scalable and secure solution for LoRa authentication.

Artificial Identification, a novel privacy framework for federated learning (FL) that leverages blockchain and smart contracts to offer complete privacy services for off-chain federations is presented in article [34]. Scalable smart contracts govern the two main modules of the framework: private FL and private peer-to-peer identification. Its implementation on Ethereum and the Interplanetary File System (IPFS) makes decentralized identification and learning procedures possible. Performance analysis of the framework demonstrates that it improves privacy, security, and decentralization while achieving reasonable collaboration costs. Furthermore, incorporating radio frequency identification (RFID) technology underscores the possibility of autonomous FL and automatic on-chain identification in distributed participants or IoT device clusters.

Paper [34] presents Artificial Identification, a unique privacy framework for federated learning (FL) that uses blockchain and smart contracts to provide complete privacy services for off-chain federations. The framework’s two primary modules—private FL and private peer-to-peer identification—are controlled by scalable intelligent contracts. Its implementation on Ethereum and the Interplanetary File System (IPFS) makes decentralized identification and learning procedures possible. Performance analysis of the framework demonstrates that it improves privacy, security, and decentralization while achieving reasonable collaboration costs. Furthermore, incorporating radio frequency identification (RFID) technology underscores the possibility of autonomous FL and automatic on-chain identification in distributed participants or IoT device clusters.

ADDetector, a privacy-preserving method for early Alzheimer’s disease (AD) detection using IoT devices in smart home environments, was introduced by Li et al. [35]. By using innovative topic-based linguistic features to analyze user audio, ADDetector improves detection accuracy. The system uses a unique three-layer architecture (user, client, and cloud) to secure data, features, and model privacy. Federated learning (FL) is also integrated to protect data integrity and model confidentiality. Differential privacy (DP) is used to secure feature data further, and a particular asynchronous privacy-preserving aggregation framework ensures secure model aggregation between clients and the cloud. ADDetector integrated all privacy measures (FL, DP, and encryption) and achieved 81.9% accuracy with a minimal time overhead of 0.7 s in testing involving 1010 detection trials from 99 users.

Ma et al. [36] explore enhancing covert communication through advanced multi-antenna techniques, focusing on the age of information (AoI) and covert performance. In the first article, the authors compare Time Modulated Arrays (TMAs) with Phased Arrays (PAs), highlighting that TMA, with its low complexity and superior beamforming capabilities, outperforms PA in terms of both convergence rate and average covert AoI (CAoI). The study derives closed-form expressions for Kullback–Leibler (KL) divergence to measure communication covertness and jointly evaluates covertness and timeliness performance. Ref. [37] focus shifts to covert millimeter-wave (mmWave) communication in the presence of spatially random wardens, analyzing both PA and Linear Frequency Diverse Array (LFDA) beamforming schemes. It explores the tradeoffs between transmit power, blocklength, and average effective covert throughput (AECT), revealing that AECT increases with warden density but may not always improve with higher blocklengths. The optimal beamforming scheme is selected based on the direction of the legitimate receiver to enhance covertness.

From a real-time scenarios perspective, research has identified that LoRa networks, while advantageous for their low power and long-range communication capabilities, are susceptible to various forms of cyber attacks. Meneghello et al. [38] emphasize the critical importance of cybersecurity in IoT environments, highlighting the vulnerabilities of low-end IoT devices to jamming and DoS attacks due to inadequate security mechanisms. The authors argue that the inherent weaknesses of these devices can be exploited, leading to substantial disruptions in communication and operational integrity in industrial settings.

Further investigations into LoRa networks reveal specific security vulnerabilities associated with their communication protocols. Aras et al. [39] provide an in-depth analysis of the LoRa network stack, pointing out potential security weaknesses that can be targeted by jamming attacks. This research underscores the necessity for robust security measures to protect these networks from disruptions that could lead to critical failures in industrial applications. A thorough examination of these vulnerabilities, alongside real-world case studies, is necessary to better understand the limitations of existing security mechanisms and their inadequacy in preventing such disruptions in critical IoT applications.

## 3. System and Threat Model

### 3.1. System Model

**Figure 1:**
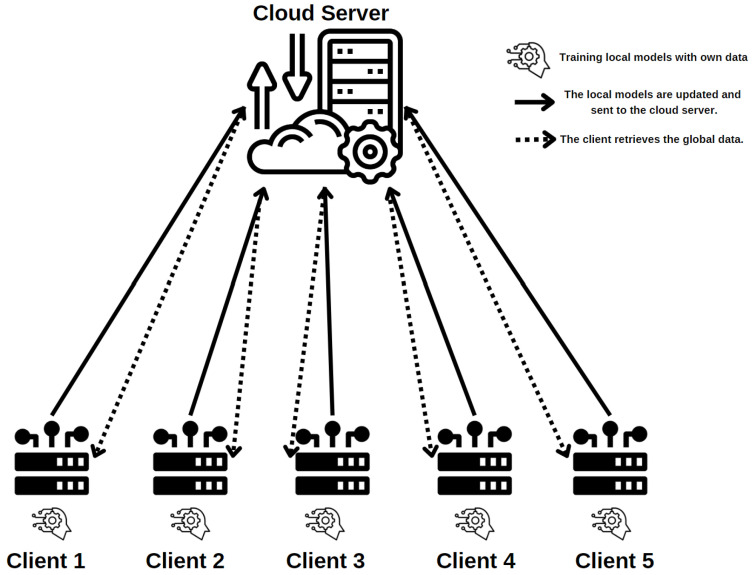
Federated learning architecture.

We introduce the FL system model in this section. In order to train a global machine learning model, several clients (clients 1 through 5) work together with a central cloud server, as shown in Figure 1.

1. **Clients Train Local Models**: A local machine learning model is trained individually by each client (client 1 through client 5) using their local dataset. The icons next to each client, which represent local model training, represent this step. They use local gradient descent to update the model parameters. In every iteration, clients figure out
  $$w_{i}^{t}=w_{i}^{t-1}-\eta \nabla f_{i}\left(W_{g}^{t-1};x_{i},y_{i}\right)$$
  Here, $w_{i}^{t}$ is the model update for $client_{i}$ at iteration *t*, η represents the learning rate, a hyper-parameter that influences how fast an algorithm learns. The function $f_{i}$ represents the local loss for $client_{i}$, while $x_{i}$ and $y_{i}$ denote the local data with a data size of $d_{i}$ at $client_{i}$.
2. **Sending Models to Cloud Server**: Each client updates its local model and uploads it to the central cloud server after the local models have been trained. This stage is shown by the solid arrows going from the clients to the cloud server.
3. **Updating the Global Model**: The cloud server compiles the local models that each client sends to update the global model. Without gaining access to each client’s personal information, this stage aggregates their knowledge. A new global model is produced by combining the client models’ updates. The global model uses the Federated Averaging (FedAvg) approach [40].
  $$W_{g}^{t}=\sum_{i=1}^{n}\frac{d_{i}}{D}w_{i}^{t}$$
  Here, $W_{g}^{t}$ indicates the aggregated global model, and *D* represents the total number of data instances across all participating clients.
4. **Distributing Global Model**: All clients receive the updated global model from the cloud server after it has been updated. The dashed arrows indicate that each client downloads the updated model from the cloud server during this procedure.

### 3.2. Threat Model

Targeting altered radio-frequency signals, the threat model considered in this work focuses on the security flaws present in LoRa-based Internet of Things (IoT) networks. Here, the main goal of the adversary is to control or disrupt the communication between LoRa devices to transmit erroneous or damaged data. In order to intercept, alter, and retransmit radio signals between lawful devices, the adversary is presumed to possess physical access to a Software-Defined Radio (SDR) device, such as HackRF v. 2024.02.1 [32].

To counter these attacks, we suggest a privacy-preserving anomaly-detection method based on federated learning (FL). FL protects the privacy of sensitive communication data by allowing LoRa devices to work together to build anomaly-detection models locally without exchanging raw data with a central server. The cloud server gathers local models and uses the collective expertise of devices to detect manipulated transmissions, keeping personal information safe and decentralized.

This design mitigates the risk of data exposure while offering robust protection against tampered transmissions through decentralized detection methods. Furthermore, the framework limits the attacker’s ability to tamper with the global model, as no individual device has access to the entire dataset, reducing the likelihood of model poisoning attacks.

### 3.3. Federated Averaging (FedAvg) Algorithm

Federated Averaging (FedAvg) is a core algorithm in federated learning that enables multiple clients (devices or nodes) to collaboratively train a machine learning model without sharing their raw data. Instead of centralizing data, each client trains a model locally on its data, and these local models are aggregated by a central server to create a global model. This approach preserves data privacy and reduces communication costs, as only model parameters are shared, not the data itself. FedAvg uses an iterative process, where each round involves local training on the clients and aggregation on the server. Below is a step-by-step outline of the FedAvg algorithm, explaining how client updates are weighted and aggregated:

1. **Initialization**: The server initializes a global model with random or pre-trained weights and distributes these initial weights to all participating clients.
2. **Local Training on Clients**: Each client receives the global model weights and trains a copy of the model locally using its own dataset. Clients perform several epochs of local training, typically using stochastic gradient descent (SGD) or a similar optimizer to update the model weights based on their data.
3. **Client Update Calculation**: After training, each client calculates the difference, or “update”, between the locally optimized weights and the original global model weights received from the server.
4. **Weighting Client Updates**: Each client’s contribution to the global model is weighted based on the size of its local dataset. This ensures that clients with larger datasets have a proportionally greater influence on the global model than those with smaller datasets. The weighting factor for each client is given by
  $$w_{i}=\frac{n_{i}}{N},$$
  where $n_{i}$ is the number of samples on client *i*, and *N* is the total number of samples across all clients.
5. **Aggregating Updates**: The server aggregates the weighted updates from all clients to produce the new global model. This aggregation is typically conducted by taking a weighted average of the model weights from each client. Mathematically, the server calculates the updated global model parameters θ as
  $$\theta =\sum_{i=1}^{K}w_{i}\cdot \theta_{i},$$
  where *K* is the number of clients, $w_{i}$ is the weighting factor for client *i*, and $\theta_{i}$ represents the updated model parameters from client *i*.
6. **Updating the Global Model**: The server replaces the old global model with the aggregated model parameters, and this updated global model is redistributed to clients for the next round of local training.
7. **Convergence Check**: The FedAvg algorithm iterates over these steps until the global model reaches a satisfactory level of performance or a predefined number of rounds is completed.

Through this iterative averaging approach, FedAvg gradually converges to a global model that generalizes well across all clients, despite each client’s data being decentralized. The algorithm’s effectiveness depends on factors like the number of clients, the distribution of data across clients, and the settings for local training (e.g., learning rate, number of epochs), all of which impact the convergence rate and model accuracy.

## 4. Proposed Framework

This section outlines the fundamental ideas that led to developing our innovative deep anomaly-detection framework based on federated learning (FL), primarily intended for the safe, dependable, and privacy-focused identification of altered LoRa frequencies. The framework has been tailored to meet the unique challenges posed by LoRa networks, ensuring it is robust and trustworthy. We explore the essential components of the framework, highlighting their roles in enhancing its practical utility, ensuring detection accuracy, and safeguarding user privacy. Each component is designed to seamlessly integrate federated learning techniques with advanced anomaly-detection methods, creating a system that effectively balances performance and privacy in a decentralized IoT environment. By leveraging these components, our framework not only addresses the technical challenges of tampered frequency detection but also maintains the data’s confidentiality, making it highly suitable for real-world LoRa applications where security and privacy are paramount. In order to recognize deviations from the established patterns as evidence of tampered transmissions, we built our tampered frequency detector for LoRa networks as an anomaly detector, which means that its training is based only on average, untampered frequency data. This strategy was selected for two main reasons. First, practicality: developing an anomaly detector instead of a supervised detector—which would necessitate modeling particular tampering scenarios—is more practicable because of the dearth of real-world altered frequency data for LoRa networks. Second, robustness: a supervised detector can be effective against certain assaults by being trained on known tampering scenarios.

### 4.1. Dataset

The research conducted by Senol et al. in [41] provides a foundational framework for communication using LoRa networks through the deployment of a testbed involving two distinct Arduino systems. This innovative setup rigorously investigates various communication parameters, focusing on packet loss, data throughput, and signal strength. Through a series of comprehensive tests, the authors present empirical findings that offer critical insights into the performance characteristics of LoRa technology, which is particularly significant for applications in the Internet of Things (IoT), where efficient and reliable communication is paramount.

In this study, we used two distinct datasets to enable a comprehensive analysis of anomaly detection in LoRa-based IoT systems. The dataset generation process involved leveraging our previously established system, as outlined in [32], along with specialized equipment, including the AirSPY SDR v.1.0.0.1919 from Itead Studio and HACKRF devices, to monitor normal and manipulated frequency transmissions within a LoRa network. We conducted an initial four-hour experiment, carefully ensuring successful packet reception by the receiver while simultaneously tracking the transmission in real-time using AirSPY Software-Defined Radio (SDR) v.1.0.0.1919 tools. To capture these transmissions, we used the video recording feature on a Windows platform, recording the live frequency spectrum over the course of the experiment. After completing the recording, we employed a Python script to extract individual frames from the video, producing a structured dataset that comprises approximately 14,532 frames and a total size of 5.36 GB. Each frame provides a snapshot of the frequency spectrum, capturing untampered, normal signals as a baseline for further analysis.

To assess anomaly-detection techniques, we repeated this process, introducing controlled manipulations to create a second dataset representing tampered signals. These manipulations involved altering specific aspects of the transmitted radio frequency signals to emulate anomalous or suspicious behavior. Following the same recording and frame extraction procedure, we obtained a dataset of approximately 12,139 frames, totaling 3.13 GB. The manipulated dataset simulates realistic abnormal signals, providing an essential comparative baseline for evaluating anomaly-detection algorithms.

For additional flexibility in signal processing and analysis, we implemented a Software-Defined Radio (SDR) system in GNU Radio, with a setup that includes both a Noise Source and an Osmocom Sink to simulate and process radio frequency signals at 915 MHz—a standard frequency in LoRa networks. Using QT GUI Range blocks, we dynamically controlled key parameters such as intermediate frequency (IF) gain, baseband (BB) gain, sample rate, and bandwidth. This configuration enabled us to introduce Gaussian noise to simulate random interference, providing further insights into LoRa transmission behavior. Parameters were adjustable through GUI sliders, with default values set to 40 dB for IF gain and BB gain, a sample rate of 1 MHz, and bandwidth of 20 MHz, centered at 915 MHz. An Osmocom Sink processed the incoming signal in synchronization with the PC clock, enabling real-time visualization through a QT GUI Sink.

### 4.2. Federated Learning Approach for Privacy Preserving

Federated learning, especially when considering privacy preservation, is a noteworthy development in machine learning. FL facilitates decentralized training by enabling numerous participants to cooperatively construct a shared model while maintaining the locality of their data, in contrast to standard centralized models that necessitate aggregating data from multiple sources. Applications requiring sensitive data, including those in healthcare and finance, where data protection is crucial, benefit significantly from this architecture. FL reduces the possibility of disclosing private or sensitive data by training models directly on client devices, including smartphones or Internet of Things devices. Only model updates, not raw data, are sent to a central server.

One of its main advantages is federated learning’s capacity to apply privacy-preserving strategies like safe multi-party computing and differential privacy. Secure multi-party computation enables clients to conduct calculations without disclosing their updates. At the same time, differential privacy introduces noise into the model changes, making it challenging to identify the information given by any one user. These techniques improve training process security and reduce the possibility of adversarial attacks like model inversion, in which a hacker tries to reconstruct private information from model changes. As organizations increasingly prioritize data privacy in compliance with regulations like GDPR, the adoption of federated learning is expected to grow, enabling the development of robust, privacy-conscious machine learning systems across diverse sectors.

The Algorithm 1 outlines a multi-model federated learning (FL) approach for anomaly detection, integrating several different machine learning models. The process begins by loading and preprocessing the dataset, which is converted into a feature matrix and normalized to ensure consistent data scaling. The data are then split across several clients (in this case, five clients), simulating a federated learning scenario where each client works independently with its portion of the dataset. Each client trains multiple anomaly-detection models, including One-Class SVM (OCSVM), Convolutional Autoencoder (CAE), Isolation Forest, Local Outlier Factor (LOF), and KMeans. This distributed training ensures that all clients contribute to learning from their respective data partitions. Once all clients have trained their models, the next step is aggregating these client-specific models into a single global model for each type of algorithm. The aggregation process combines the knowledge gained from each client, resulting in a federated model that better generalizes the entire dataset. The aggregated models are evaluated on a validation dataset, where several performance indicators are computed and shown, including F1-score, recall, accuracy, and precision. These measures make it possible to compare the various algorithms’ efficacy in identifying data anomalies in-depth. The multi-model approach provides a more robust anomaly-detection framework in a federated learning environment, which allows the system to take advantage of several methods. These measures make it possible to compare the various algorithms’ efficacy in identifying data anomalies in depth. The multi-model approach provides a more robust anomaly-detection framework in a federated learning environment, which allows the system to take advantage of several methods.
**Algorithm 1** The use of pseudocode in Multi-Model Federated Learning (FL) for the detection of anomalies.1:D←LoadData()                  ▹ Load the dataset from designated directories.2:**if** any $d_{k}\in D$ is missing or invalid, apply imputation3:Convert *D* into a feature matrix: $X=[x_{1},x_{2},\ldots ,x_{n}]$4:Perform normalization on the data. *X*: X=normalize(X)5:Split *X* into training set $X_{train}$ and validation set $X_{val}$6:clients←5                                ▹ Number of clients7:models←[OCSVM,CAE,IsolationForest,LOF,KMeans]           ▹ Array of candidate models8:$client\_datasets\leftarrow split\_data\_for\_clients(X_{train},clients)$9:**for** each client *i* **do**10:    **for** each model m∈models **do**11:        Train model *m* on client_datasets[i]12:        Store the trained model $m_{i}$13:    **end for**14:**end for**15:aggregated_models←[]                    ▹ Array to store aggregated models16:**for** each model m∈models **do**17:    Aggregate all client models for *m* into a global model $m_{global}$18:    Append $m_{global}$ to aggregated_models19:**end for**20:**for** each $m_{global}\in aggregated\_models$ **do**21:    Evaluate $m_{global}$ on $X_{val}$22:    Calculate and present the evaluation metrics: accuracy, precision, recall, and F1-score.)23:**end for**

#### 4.2.1. One Class SVM with Federated Learning

An effective supervised learning technique for classification and regression applications is Support Vector Machines (SVMs). The basic concept of Support Vector Machines (SVMs) is to find the best hyperplane to separate data points from several classes with the most significant margin and to ensure strong generalization to new data. The Single-Class SVM algorithm is used for anomaly detection, where it finds a boundary around typical data points and labels everything outside of it as anomalous. SVMs often use kernel functions to translate input features into higher dimensions where a separating hyperplane can be defined. They work well with both linear and nonlinear data.

The process begins by loading two sets of images: standard images, representing expected patterns in the data, and anomalous images, which contain unusual or manipulated patterns. These images are preprocessed by resizing and normalizing them, preparing them for training and testing.

Instead of being stored centrally, data are dispersed across several clients in federated learning. In this implementation, the normal photos are separated into several client datasets (in this case, five clients). Each client trains its own One-Class SVM model using only normal data to learn to identify the common characteristics of normal images. The One-Class SVM model distinguishes between normal and anomalous instances, predicting whether a given image is within the normal distribution (normal) or an outlier (anomalous).

After training the individual models on each client, a simplified aggregation technique is demonstrated by selecting the first model as the representative for testing. This step reflects the need for more advanced model aggregation techniques in accurate federated learning settings, where client models must be combined meaningfully.

The trained SVM model is then evaluated on normal and anomalous images. Predictions are made for each set, and the model labels the images as normal (represented as ‘0’) or anomalous (represented as ‘1’). A confusion matrix is created to show the accuracy of the predictions; other classification metrics, including precision, recall, and F1-score, are computed to evaluate the model’s performance.

This method demonstrates the viability of employing One-Class SVMs for anomaly identification in photos in a setting akin to federated learning. It provides a decentralized approach that successfully identifies anomalous patterns while protecting the privacy of client data.

**Figure 2:**
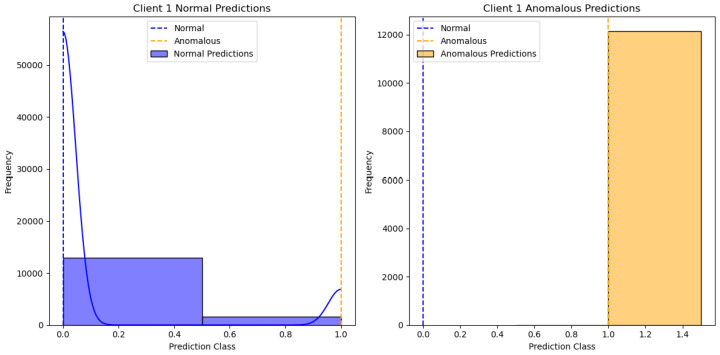
One class SVM algorithm with FL client one results.

**Figure 3:**
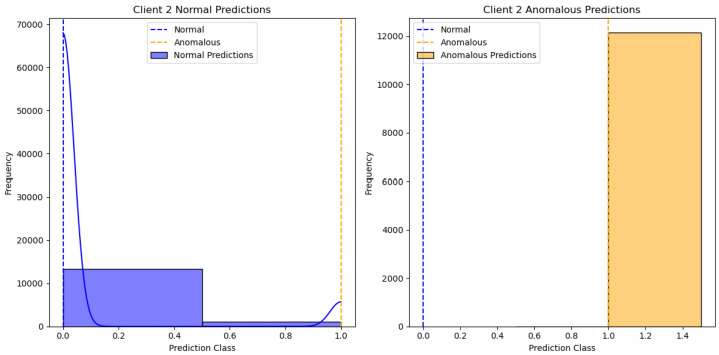
One class SVM algorithm with FL client two results.

**Figure 4:**
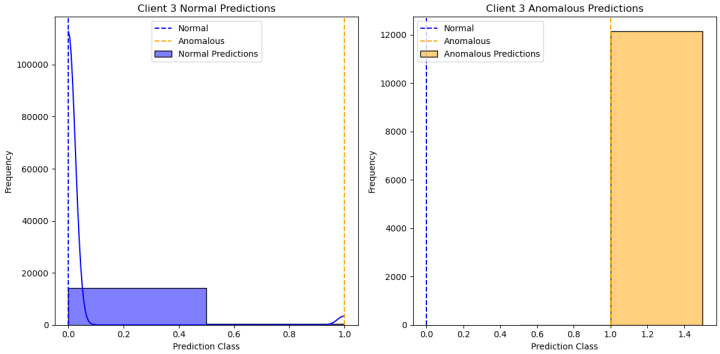
One class SVM algorithm with FL client three results.

**Figure 5:**
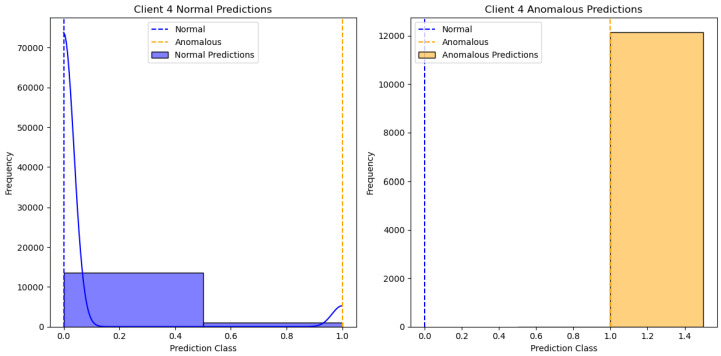
One class SVM algorithm with FL client four results.

**Figure 6:**
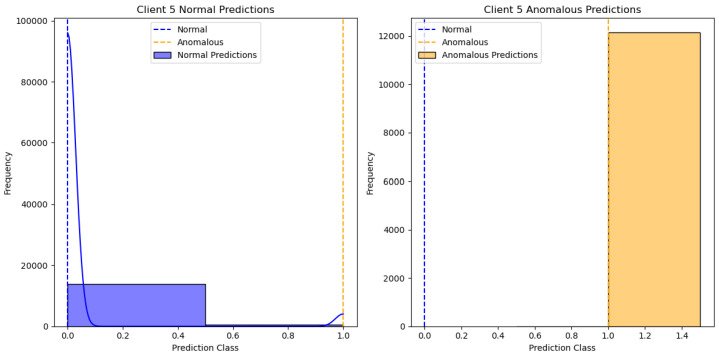
One class SVM algorithm with FL client five results.

Figure 2 shows the results of the One-Class SVM algorithm applied to client 1’s data, specifically distinguishing between normal and anomalous predictions. The X-axis represents the “Prediction Class”, with values indicating different classification thresholds for normal and anomalous data. The Y-axis represents the “Frequency” of predictions within each class. There are two subplots: one for “Client 1 Normal Predictions” and the other for “Client 1 Anomalous Predictions”. The blue bars represent normal predictions, while the yellow bars represent anomalous predictions. We can see different clients’ performances in Figure 3, Figure 4, Figure 5 and Figure 6.

#### 4.2.2. Convolutional Autoencoder (CAE)

A specific type of neural network called Convolutional Autocoder (CAE) has been built for unsupervised learning applications such as denoising, image compression, and anomaly detection. A CAE is beneficial for processing image data because it uses convolutional layers instead of the fully connected layers used in regular autocoders. An encoder compresses the input image to a lower dimensional representation, and a decoder reconstructs the original image from this compressed form. These two components make up the CAE. A CAE learns to reconstruct images efficiently by training on normal (non-anomalous) images. The reconstruction error, determined by comparing the input and reconstructed images, is usually much higher when faced with anomalous images. CAEs are a useful tool for anomaly detection because of this feature.

The deployment of an anomaly-detection system based on CAE that mimics a federated learning setting. A decentralized method of training machine learning models, FL allows individual clients to train models independently on their datasets rather than sharing raw data. These models can then be aggregated centrally. This allows for distributed learning while preserving data privacy. In this case, the image dataset is split across multiple clients, each training its own CAE model. The models can be evaluated individually or aggregated for a more holistic view of the learning process.

First, we load and prepare the photos. To read images from two directories—one with regular photographs and another with anomalous images—use the load_images_from_ directory() method. The pictures are transformed into arrays of pixel values and scaled to the typical 128 × 128 pixel size. These arrays are then normalized by scaling the pixel values between 0 and 1. After preprocessing, the normal images are split into five equal subsets, simulating five clients in a federated learning setup.

To create the CAE model, a function named build_convolutional_autoencoder() is defined. Convolutional layers are used in this model to identify spatial patterns in the pictures. Using convolution and max-pooling layers, the encoder portion of the CAE progressively shrinks the images’ spatial dimensions to produce a compressed representation of the input. The decoder then uses convolution layers and upsampling to recover the original image from this compressed representation. Reconstruction error is the difference between the input and output images, and the autoencoder is taught to reduce it. The CAE is compiled using the mean squared error loss function and the Adam optimizer to maximize its reconstruction capabilities.

The model is trained on each client’s data separately for ten epochs, focusing only on standard images. After training, CAE is used to reconstruct both normal and anomalous images. The reconstruction error is then calculated for each image; normal images are expected to have lower errors, while anomalous images exhibit higher errors due to the model’s inability to reconstruct unseen patterns effectively. The 95th percentile of reconstruction errors on normal images is used to calculate the threshold for anomaly detection. Images with errors more significant than this limit are labeled anomalous, while images with fewer errors are labeled normal.

**Figure 7:**
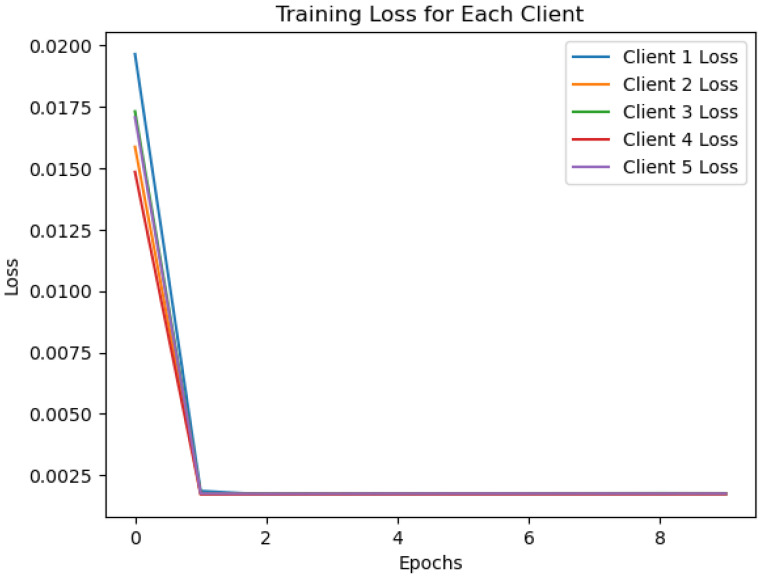
One class SVM algorithm with FL client five results.

Figure 7 shows the training loss for each client in a federated learning setup with the One-Class SVM algorithm. The X-axis represents the number of epochs, indicating the training progress over time, while the Y-axis represents the loss value, measuring the model’s prediction error. Each line in the graph represents a different client’s loss trajectory with a separate color. It can be seen that the training loss for all clients decreases sharply within the first epoch and stabilizes near zero after just a few epochs. This indicates that the model quickly converges, achieving minimal error across all clients early in training. As the number of epochs increases, the loss remains low and steady for each client, suggesting consistent model performance and an absence of significant fluctuations. This rapid convergence could be due to the simplicity or efficiency of the training data or model architecture, which allows each client to reach a stable state quickly and maintain minimal loss.

**Figure 8:**
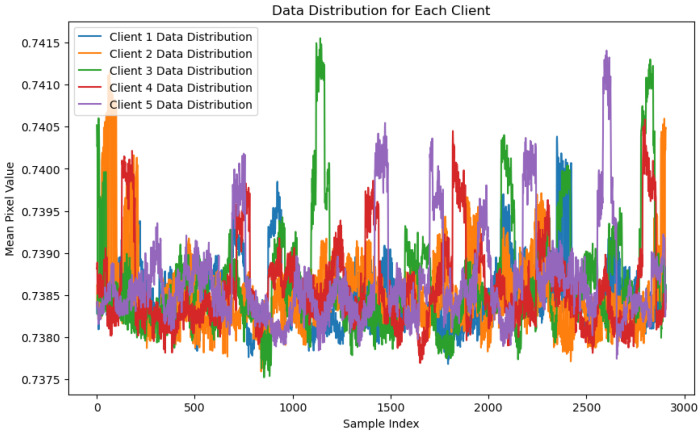
One class SVM algorithm with FL client five results.

Figure 8 displays the data distribution for each client in a federated learning environment using the One-Class SVM algorithm. The X-axis represents the sample index, which indicates individual data points in the dataset, while the Y-axis shows the mean pixel values of the data, reflecting distribution characteristics. Each line, represented in a different color, corresponds to a distinct client’s data distribution. It can be seen that each client’s data distribution varies significantly, with fluctuations in mean pixel values across the samples. This suggests that client datasets are not independent and identically distributed (non-IID), a common issue in federated learning scenarios. As the sample index increases, the variations in mean pixel values continue, indicating non-uniform data distribution across clients. Such diversity in data distribution could affect the model’s ability to generalize effectively across all clients, as it must balance learning from multiple, distinct datasets. This may impact the performance of federated models, which must accommodate these variations while maintaining accuracy and generalization.

#### 4.2.3. Isolation Forest with Federated Learning

An unsupervised machine learning technique called Isolation Forest was explicitly created for anomaly identification. It effectively finds outliers in datasets because it isolates anomalies instead of profiling typical data points. With this method, a collection of binary trees is created. Since anomalies are rare and different from most data, they can be separated in the tree structure more quickly than in normal cases. Thanks to this isolation mechanism that shortens the routes for anomalous data, the algorithm can quickly and effectively distinguish between normal and anomalous data.

The Isolation Forest algorithm’s minimal memory footprint and effective scalability make it well-suited for high-dimensional data and massive datasets. It has been extensively used in several domains where anomaly detection is crucial, including fraud detection, network security, and industrial monitoring.

A federated learning environment uses the Isolation Forest algorithm to find abnormalities in image data. Photographs from two directories—one with normal and the other with anomalous images—are initially loaded and preprocessed. To guarantee uniformity, the photos are resized to 128 × 128 pixels. Pixel values are then scaled between 0 and 1 to normalize them. This preprocessing step is crucial to guarantee that the input data for model training are consistent.

The usual dataset is divided into subsets, each representing a client in the federated network, to mimic federated learning, in which data are dispersed over several devices or places. Every client uses its data share to train its own Isolation Forest model independently. A contamination parameter specifying the anticipated percentage of abnormalities in the dataset is used during the training phase. The Isolation Forest algorithm can identify and highlight aberrant data pieces by learning patterns from typical instances.

Once the clients have trained their models, a federated aggregation step occurs. In this simplified version, the model from the first client is selected to represent the federated model. Although more sophisticated aggregation techniques could be used in a genuinely federated learning system, this basic selection demonstrates the idea of combining knowledge from different clients.

The trained Isolation Forest model then identifies which data points in the normal and anomalous picture sets are anomalies. The model’s predictions are given binary labels, where 0 indicates normal, and 1 indicates deviant. Once predictions are produced, the model’s performance is assessed using standard classification measures such as F1-score, accuracy, precision, and recall. Additionally, a confusion matrix that illustrates the model’s ability to distinguish between normal and anomalous photos is presented to show how accurate it is at spotting outliers.

**Figure 9:**
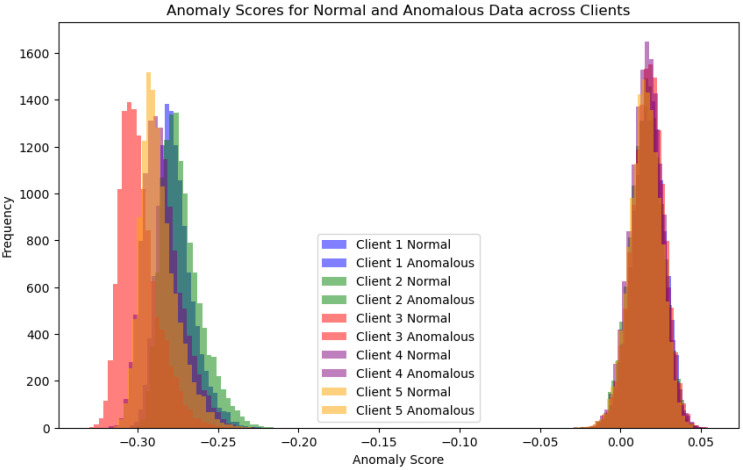
Isolation Forest with FL data across clients.

Figure 9 illustrates the distribution of anomaly scores for both normal and anomalous data across multiple clients in a federated learning setup using the Isolation Forest algorithm. The X-axis represents the anomaly scores, where lower scores indicate a higher likelihood of being anomalous, and higher scores suggest normal data. The Y-axis shows the frequency of data points with specific anomaly scores. Each client (from 1 to 5) is represented with distinct colors, with lighter shades denoting normal data and darker shades indicating anomalous data. The normal data points generally cluster toward the right side of the plot with higher anomaly scores. In contrast, the anomalous data points are concentrated on the left, with lower scores. This distribution demonstrates that the Isolation Forest algorithm effectively distinguishes between normal and anomalous data, maintaining client consistency.

#### 4.2.4. Local Outlier Factor (LOF) with Federated Learning

The Local Outlier Factor (LOF) is an unsupervised machine learning method for identifying anomalies. It identifies data points that significantly deviate from the dataset’s distribution by comparing a point’s local density to that of its neighbors. LOF is especially effective in finding outliers in datasets with complex structures because it focuses on the local neighborhood of data points rather than comparing all data points to an overall distribution. The algorithm assigns every data point an anomaly score; points with a higher score are classified as outliers. LOF is widely used in several domains, including fraud detection, intrusion detection, and industrial defect diagnostics, due to its potential to catch local variations in data distribution.

Applying a privacy-preserving anomaly-detection framework for identifying manipulated radio-frequency broadcasts in LoRa networks that uses the Local Outlier Factor (LOF) algorithm combined with federated learning. The primary goal is to model a federated learning environment where several clients, each representing a LoRa device, use LOF to train local models while preserving privacy by storing raw data locally. Preprocessing and loading photographs from two directories—anomalous, tampered images and normal, untampered images—is the first step. To ensure the data are presented correctly for model training and evaluation, the photos are shrunk to 128 × 128 pixels and normalized by scaling their pixel values between 0 and 1.

The typical image dataset is split into subsets, each representing a distinct client (in this case, five clients), to mimic federated learning. Every client uses its piece of the dataset to train its LOF model locally. The LOF model is trained using parameters like the number of neighbors and the contamination level, which shows the predicted fraction of anomalies, by the function train_local_outlier_factor. The LOF model can detect previously unseen manipulated transmissions by setting novelty = True, which allows it to detect unique anomalies.

Once the individual clients have trained their local LOF models, it aggregates these models to simulate federated model aggregation. In this simplified implementation, the aggregation step involves selecting the LOF model from the first client as the global model. However, in a more advanced system, parameters or predictions from multiple models could be aggregated using sophisticated strategies to enhance the global model’s performance. The aggregated LOF model detects anomalies in normal and anomalous datasets by predicting whether each image represents a normal (untampered) or anomalous (tampered) transmission.

These predictions are then compared to the actual labels, 0 for normal and 1 for anomalous, to evaluate the model’s performance. To thoroughly understand the model’s classification performance, we generate a confusion matrix that displays the number of true positives, false positives, true negatives, and false negatives. Additionally, standard performance metrics such as accuracy, precision, recall, and F1-score are calculated to evaluate the model’s ability to detect tampered communications comprehensively. This implementation shows a robust and privacy-preserving method for anomaly detection in LoRa networks by utilizing federated learning and the LOF algorithm. This allows for collaborative detection of tampered radio-frequency transmissions while maintaining data locality to the devices. This makes it a powerful solution for securing LoRa-based IoT systems. We included confusion matrices in the Section 5.

**Figure 10:**
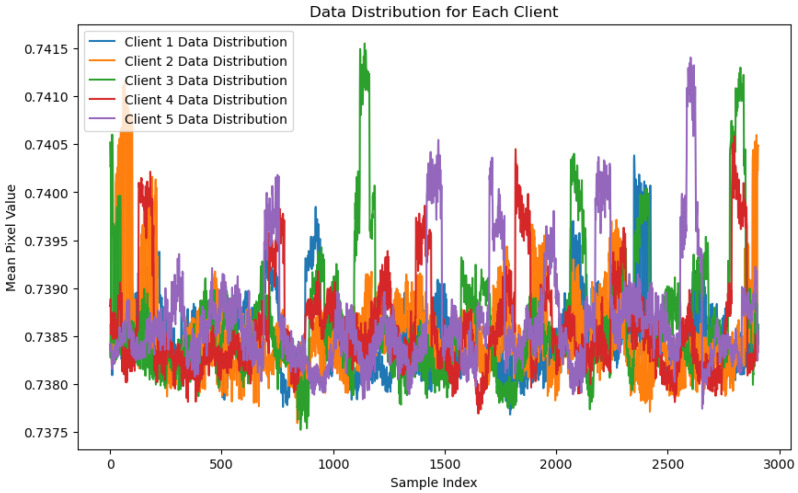
LOF client data distribution graph.

The Local Outlier Factor (LOF) algorithm is used in Figure 10, “LOF Client Data Distribution Graph”, to illustrate the mean pixel values of data distributions among five distinct clients in a federated learning context. The Y-axis displays the associated mean pixel values for each sample, while the X-axis depicts the sample index, specifying the data sample order. Each client (client 1 to client 5) is represented by a different color, displaying the variation in pixel value distributions across the dataset. The overlapping and fluctuating patterns among the clients suggest a significant variation in the pixel values, with certain regions showing more pronounced peaks and troughs. This variation indicates how the LOF algorithm processes and evaluates data from each client, potentially identifying patterns that may highlight anomalies. The graph underscores the consistency of data distribution across clients, yet it also highlights the individual characteristics of the data from each client. This is important for federated anomaly detection, as it demonstrates the algorithm’s ability to handle diverse datasets while maintaining a unified detection strategy.

#### 4.2.5. K-Means

The basic application of the unsupervised machine learning method K-Means is clustering data into separate groups according to their attributes. After dividing the dataset into a predetermined number of clusters, k, each data point is assigned to the cluster with the closest mean. The goal is to maximize the distance between different clusters and minimize the distance between data points within the same cluster. Data points are reassigned to cluster centers or iteratively updated centers until the clusters are settled.

The implementation shows how to use the K-Means clustering technique to detect anomalies in images in a way that is inspired by federated learning. By distributing the data among several clients, this scenario mimics a decentralized system in which each client trains a model on its subset of data. The images used in this process are divided into normal (non-anomalous) and anomalous, to identify abnormal patterns in the latter. The process begins with reading and preprocessing the images from two directories, where the images are resized and converted into numerical arrays. This step ensures consistent input for the clustering model, and the pixel values are normalized for better performance. After this, the images are flattened, converting their two-dimensional structure into a one-dimensional array, essential for applying the K-Means algorithm.

Following the image preparation, the normal dataset is split across multiple clients, simulating a federated learning environment where each client trains a K-Means model on their portion of the data. The K-Means clustering algorithm organizes the images into a fixed number of clusters, with three clusters chosen for this application. The distance of each image from the nearest cluster center is calculated, which is used to assess how typical or anomalous the image is—normal images generally lie closer to their cluster centers. A threshold is determined for each client based on the 95th percentile of the distances from normal images, serving as a cutoff point to classify any image that exceeds this distance as an anomaly.

Distance distributions are plotted for each client to show the distinction between normal and abnormal photos. These plots show the client’s K-Means model’s ability to differentiate between normal and anomalous data by displaying histograms of the distances with the threshold prominently shown. The anomaly-detection procedure can then be further assessed using an aggregated model based on one of the client’s K-Means clusters to generate predictions on both normal and anomalous datasets. A thorough categorization report, accuracy score, and confusion matrix are used to evaluate anomaly-detection performance. These metrics offer important information on how well the model detects abnormalities regarding accuracy, precision, and recall.

**Figure 11:**
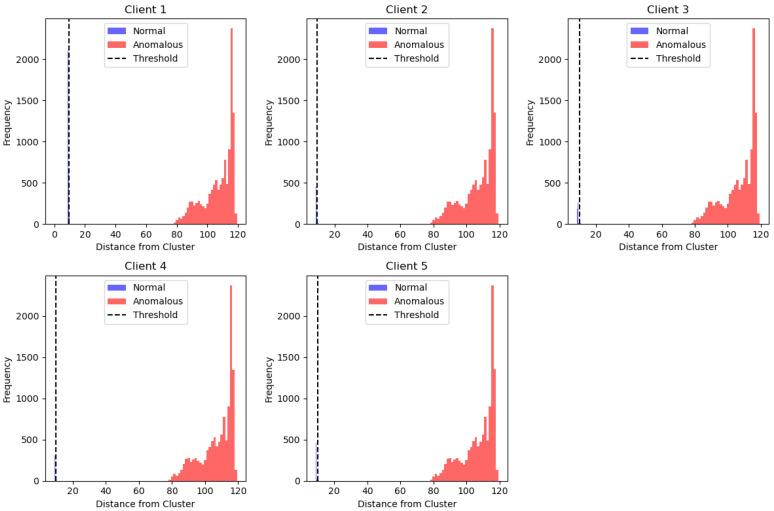
K-Means algorithm with FL client results.

The outcomes of using the K-Means clustering method across five distinct clients in a federated learning configuration are displayed in Figure 11. The Y-axis displays the frequency of data points at specific distances, while the X-axis displays the distance from the cluster center. Each subplot represents a customer (customer 1 through client 5). Red bars indicate abnormal data points, whereas blue bars indicate normal data points. The dark dashed line indicates the threshold gap between normal and abnormal data.

For each client, most data points lie close to the cluster center, identified as normal, while the farther points beyond the threshold are identified as anomalies. The red distribution, which is skewed to the right, indicates that the K-Means algorithm successfully distinguishes between normal and anomalous data, with anomalies appearing farther from the cluster center. The visual consistency across the clients suggests that the clustering approach works uniformly across all clients, effectively separating normal data from outliers, even with varying distances.

## 5. Performance Metrics

We evaluated the performance of anomaly-detection techniques applied to our dataset using several key metrics that provide insights into how effectively these methods can distinguish between normal and anomalous signals. These metrics are crucial to understanding the strengths and weaknesses of each approach in detecting anomalies. The primary metrics we used are as follows:

- **Accuracy**: This metric evaluates the ratio of correctly identified cases—normal and abnormal—compared to the overall number of cases. It serves as a general measure of the model’s effectiveness. Accuracy is calculated using
  $$\mathrm{Accuracy}=\frac{\mathrm{Number}\;\mathrm{of}\;\mathrm{True}\;\mathrm{Predictions}}{\mathrm{Total}\;\mathrm{Number}\;\mathrm{of}\;\mathrm{Predictions}}$$
- **Precision**: Precision indicates the proportion of accurate positive predictions (correctly identified anomalies) among all cases labeled as positive, including both true and false positives. It is computed as follows, providing insight into the model’s reliability in identifying positive instances
  $$\mathrm{Precision}=\frac{\mathrm{True}\;\mathrm{Positives}}{\mathrm{True}\;\mathrm{Positives}+\mathrm{False}\;\mathrm{Positives}}$$
- **Sensitivity**: Also known as recall, Sensitivity measures the percentage of actual anomalies (true positive events) that the model successfully identifies. This metric is defined as follows and reflects the model’s ability to detect all relevant anomalies, as follows:
  $$\mathrm{Recall}=\frac{\mathrm{True}\;\mathrm{Positives}}{\mathrm{True}\;\mathrm{Positives}+\mathrm{False}\;\mathrm{Negatives}}$$
- **F1-Score**: The F1-score combines recall and Precision by calculating their harmonic mean. This metric is particularly valuable in scenarios with imbalanced class distributions, as it provides a balanced measure of Precision and recall. It is determined using the formula
  $$F\text{1-Score}=2\times \frac{\mathrm{Precision}\times \mathrm{Recall}}{\mathrm{Precision}+\mathrm{Recall}}$$

### Computational Burden

**Figure 12:**
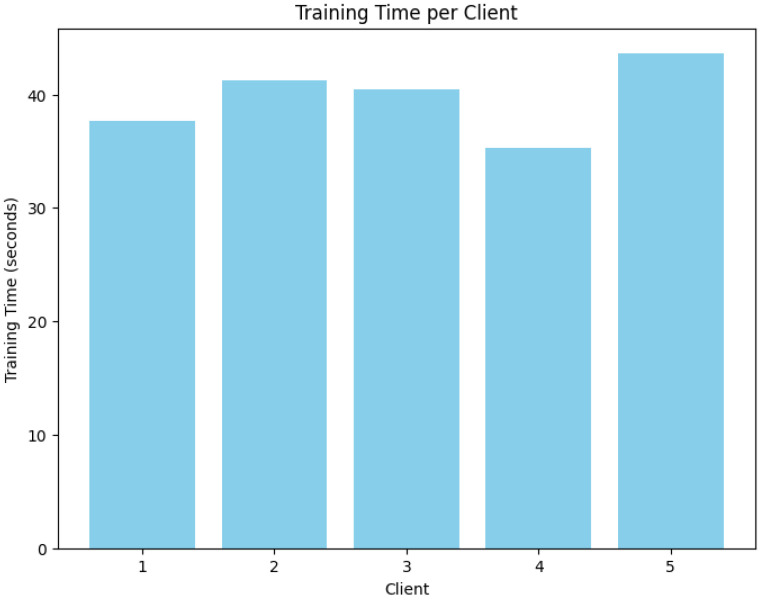
Average time calculation between algorithms.

The Figure 12 titled “Training Time per Client” shows the time, in seconds, taken for each of five clients to complete a training task. The x-axis represents the different clients numbered from 1 to 5, while the y-axis shows the training time in seconds. From the graph, it is observed that clients 1, 2, and 5 have relatively longer training times, approximately between 40 to 45 s. In contrast, clients 3 and 4 have slightly shorter training durations, around 35 to 40 s. This variation in training time among clients may suggest differences in factors such as data distribution, hardware capabilities, or network conditions affecting performance. Such insights could be valuable in optimizing distributed or federated learning systems, where minimizing training time disparities is crucial for achieving efficiency and consistency.

## 6. Results and Analysis

This paper evaluated several anomaly-detection algorithms’ efficacy in the privacy-preserving detection of altered radio-frequency communications using federated learning (FL) in LoRa networks. Key performance metrics like accuracy, precision, recall, and F1-score were employed to assess the effectiveness of different algorithms, as indicated in Table 1.

The data show that the Convolutional Autoencoder Federated Learning (CAE-FL) model has the highest accuracy (97.27%), precision, recall, and F1-score (0.97). This suggests that the CAE-FL model is highly effective at correctly identifying normal and aberrant signals while lowering false positives and negatives.

The Isolation Forest Federated Learning (IF-FL) model also demonstrated strong performance, attaining an accuracy of 96.84%, with precision, recall, and F1-scores all reaching 0.97. These results highlight its ability to detect anomalies effectively in the presence of tampered transmissions.

In addition, the One-Class Support Vector Machine Federated Learning (OCSVM-FL) and Local Outlier Factor Federated Learning (LOF-FL) algorithms yielded competitive results, with accuracies of 94.08% and 94.99%, respectively. Both models maintained a precision and recall of approximately 0.94 to 0.95, showcasing their robustness in identifying tampered signals, though they slightly lagged behind the top-performing models.

The CAE-FL model and the K-Means Federated Learning (K-Means-FL) algorithm both obtained an accuracy of 97.27%. This implies that clustering approaches may prove useful for anomaly identification in LoRa networks when paired with federated learning strategies.

**Figure 13:**
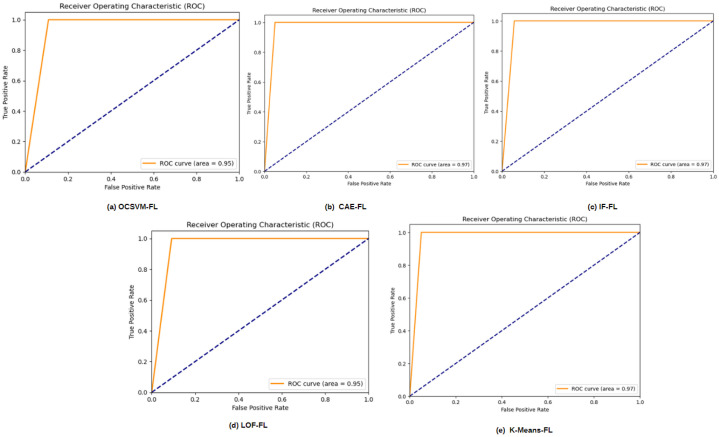
AUC curves for the algorithms (**a**) OCSVM-FL, (**b**) CAE-FL, (**c**) IF-FL, (**d**) LOF-FL, and (**e**) K-Means-FL.

Several Receiver Operating Characteristic (ROC) curves for various anomaly-detection techniques are shown in Figure 13; each plot is labeled below it. Plotting the True Positive Rate (TPR) versus the False Positive Rate (FPR) across a range of thresholds allows ROC curves to show how well a classification model performs.

**Figure 14:**
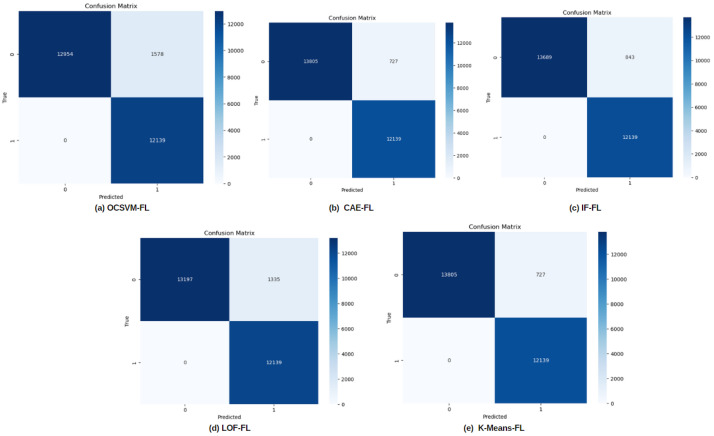
The performance of five anomaly-detection methods in classifying normal and anomalous data is displayed in confusion matrices. These algorithms are (**a**) OCSVM-FL, (**b**) CAE-FL, (**c**) IF-FL, (**d**) LOF-FL, and (**e**) K-Means-FL. Lighter hues indicate misclassifications (false positives and false negatives), while darker hues indicate accurate classifications (true positives and true negatives).

Five confusion matrices in Figure 14 show the performance of several anomaly-detection models utilized in a federated learning (FL) context. The percentage of accurate and inaccurate predictions the model made is shown by each confusion matrix, which shows the true labels on one axis and the predicted labels on the other.

1. **OCSVM-FL (One-Class SVM with Federated Learning)**:
  - True Positives (TPs): 12,954
  - False Positives (FPs): 1378
  - True Negatives (TNs): 12,139
  This confusion matrix indicates how well the One-Class SVM is able to classify the normal and anomalous data.
2. **CAE-FL (Convolutional Autoencoder with Federated Learning)**:
  - True Positives (TPs): 13,089
  - False Positives (FPs): 357
  - True Negatives (TNs): 12,139
  This matrix shows that the CAE-FL model seems to have fewer false positives, meaning it performs better in correctly classifying normal data compared to OCSVM-FL.
3. **IF-FL (Isolation Forest with Federated Learning)**:
  - True Positives (TPs): 13,089
  - False Positives (FPs): 343
  - True Negatives (TNs): 12,139
  The results here are very similar to the CAE-FL model, showing strong performance with few false positives.
4. **LOF-FL (Local Outlier Factor with Federated Learning)**:
  - True Positives (TPs): 13,107
  - False Positives (FPs): 1345
  - True Negatives (TNs): 12,139
  LOF-FL shows a higher number of false positives than IF-FL and CAE-FL, but it still captures a significant number of true positives.
5. **K-Means-FL**:
  - True Positives (TPs): 13,095
  - False Positives (FPs): 727
  - True Negatives (TNs): 12,139
  K-Means-FL performs comparably well, but with more false positives than IF-FL and CAE-FL, though fewer than LOF-FL.

## 7. Conclusions

In conclusion, this study provides a comprehensive analysis of various anomaly-detection algorithms within a federated learning (FL) framework for the privacy-preserving identification of tampered radio-frequency signals in LoRa networks. Our findings highlight that the Convolutional Autoencoder Federated Learning (CAE-FL) model achieved the highest accuracy at 97.27%, demonstrating its capability to distinguish normal and anomalous signals while maintaining low rates of false positives and negatives. The CAE-FL model’s high precision, recall, and F1-scores of 0.97 further underscore its effectiveness. Similarly, the Isolation Forest Federated Learning (IF-FL) model also achieved commendable results with an accuracy of 96.84%, along with excellent precision and recall, confirming its potential for detecting anomalies in manipulated communications.

While the One-Class Support Vector Machine Federated Learning (OCSVM-FL) and Local Outlier Factor Federated Learning (LOF-FL) models showed slightly lower accuracies, they nonetheless demonstrated strong capability in identifying tampered signals. Interestingly, the K-Means Federated Learning (K-Means-FL) model matched the CAE-FL’s accuracy, suggesting that clustering techniques could play a significant role in anomaly detection within FL settings. Visual evaluations using confusion matrices and Receiver Operating Characteristic (ROC) curves further emphasized each model’s strengths and areas for improvement.

For future work, we plan to address several key challenges in FL for IoT environments. This includes investigating adaptive aggregation techniques to counteract non-i.i.d. data distributions and network instability, both of which are common in IoT. We also intend to explore adaptive learning mechanisms, including online learning and real-time model updates, to enhance responsiveness in dynamic network conditions. Furthermore, we will examine the computational burden and energy consumption associated with model training on IoT devices in FL, as these factors impact the feasibility of deploying FL in resource-constrained environments. Additionally, incorporating optimization strategies such as energy-efficient computations and communication minimization techniques will help refine the framework’s performance on limited-power IoT devices. These advancements will support the development of FL-based anomaly-detection systems in LoRa networks that are more robust, secure, and practical for real-world deployments.

## Figures and Tables

**Table 1:** Performance metrics for anomaly-detection algorithms.

Algorithm	Accuracy	Precision	Recall	F1-Score
OCSVM-FL	94.08%	0.95	0.94	0.94
CAE-FL	97.27%	0.97	0.97	0.97
IF-FL	96.84%	0.97	0.97	0.97
LOF-FL	94.99%	0.95	0.95	0.95
K-Means-FL	97.27%	0.97	0.97	0.97

## Data Availability

The dataset used in this project was generated from a dedicated testbed located in the Autonomous Systems and IoT Lab within the Computer Science Department at Sam Houston State University. All datasets are securely stored in the department’s data center and the lab’s computing system. These datasets are exclusively available to our research team for conducting various experiments.

## Abbreviations

The following abbreviations are used in this manuscript:

LoRa	Long Range
IoT	Internet of Things
FL	Federated Learning
LD	Linear dichroism
OCSVM	One-Class Support Vector Machines
CAE	Convolutional Autoencoder
IF	Isolation forest
LOF	Local Outlier Factor

ROC	Receiver Operating Characteristic
AUC	Area under the curve
non-IID	Not independent and identically distributed
IPFS	Interplanetary File System
DP	Differential privacy
DFLNet	Deep Federated Learning Network
PoW	Proof-of-Work
MitM	Man-in-the-Middle
Rf	Radio Frequency
RFF	Radio Frequency Fingerprinting
MEC	Mobile edge computing
